# Value of serum miR-21, HE4 and CA125 in surveillance for postoperative recurrent or metastatic ovarian cancer

**DOI:** 10.12669/pjms.38.4.5158

**Published:** 2022

**Authors:** Zhenying Gong, Sugui Han, Chunlei Zhang, Honghuan Zhao, Jinxia Xu, Xing Sun

**Affiliations:** 1 Zhenying Gong, Department of Obstetrics and Gynecology, Luanzhou Hospital of Traditional Chinese Medicine, Tangshan 063000, Hebei, China; 2 Sugui Han, Department of Clinical Laboratory, Tangshan People’s Hospital, Tangshan 063000, Hebei, China; 3 Chunlei Zhang, Department of Clinical Laboratory, Tangshan People’s Hospital, Tangshan 063000, Hebei, China; 4 Honghuan Zhao, Department of Clinical Laboratory, Tangshan People’s Hospital, Tangshan 063000, Hebei, China; 5 Jinxia Xu, Department of Clinical Laboratory, Tangshan People’s Hospital, Tangshan 063000, Hebei, China; 6 Xing Sun, Department of Clinical Laboratory, Tangshan People’s Hospital, Tangshan 063000, Hebei, China

**Keywords:** miR-21, Human epididymal secretory protein 4, Carbohydrate antigen 125, Ovarian cancer, Recurrence and metastasis

## Abstract

**Objectives:**

To study the value of serum miR21, human epididymal secretory protein 4 (HE4) and carbohydrate antigen 125 (CA125) in the surveillance for postoperative recurrent or metastatic ovarian cancer.

**Methods:**

A total of 169 patients diagnosed with ovarian conditions in Luanzhou Hospital of Traditional Chinese Medicine during January 2016 and March 2019 were divided into a benign lesion (BL) group and an ovarian cancer (OC) group by pathological findings and assigned to a good prognosis (GP) group and a poor prognosis (PP) group according to the follow-up results. A real-time fluorescence quantitative PCR (RT-fqPCR) system was utilized to detect the serum level of miR-21; an enzyme-linked immunosorbent assay (ELISA) was conducted to determine the serum level of HE4; electrochemiluminescence (ECL)-based imaging analysis was performed to measure serum CA125. A receiver operating characteristic (ROC) curve was depicted to analyze the predictive value of serum miR-21, HE4, and CA125 for poor postoperative prognosis in patients with ovarian cancer.

**Results:**

Compared with the control group, the BL and OC groups had substantially elevated expression of miR-21, HE4, and CA125 in serum, and the serum levels of miR-21, HE4, and CA125 in the OC group were significantly higher than in the BL group. In the OC group, the serum levels of miR-21, HE4, and CA125 were independent of age and pathological patterns and associated with the clinical staging, degree of transformation and lymphatic metastasis of ovarian cancer; after laparoscopic ovarian tumorectomy, the serum levels of miR-21, HE4, and CA125 were markedly reduced in comparison with the preoperative levels. Compared with the GP group, the PP group experienced a dramatic increase in serum miR-21, HE4, and CA125 expression. The ROC curve showed that the detection of miR-21, HE4, and CA125 was a highly sensitive and specific method to predict the poor prognosis in ovarian cancer; a patient with ovarian cancer was at high risk of a poor prognosis when the serum levels of miR-21, HE4, and CA125 exceeded 1.536, 157.004 pmol/L and 175.243 kU/L, respectively, in which case early intervention should be made to prevent recurrent or metastatic ovarian cancer.

**Conclusion:**

Elevated expression of miR-21, HE4, and CA125 in serum is closely associated with the disease status of ovarian cancer. Therefore, the simultaneous detection of these tumor markers has some diagnostic value for postoperative recurrence and metastasis of ovarian cancer.

## INTRODUCTION

Ovarian cancer is a common malignancy of the female reproductive system, which has an incidence rate lower than corpus cancer and endometrial cancer and is usually seen in women at the age of about 50 years old. Ovarian cancer causes no detectable symptoms during the early stage. However, it can progress quickly and metastasize rapidly. In most cases, ovarian cancer has already progressed to an advanced stage at the time of onset, with the patients presenting such symptoms as bloating, abdominal pain and weight loss.^1^,^2^

Tumorectomy is currently the standard treatment for ovarian cancer, which however entails a relatively high risk of postoperative recurrence and metastasis that undermines the quality of life for patients with ovarian cancer.^3^ Therefore, it is important to evaluate the risk of postoperative recurrence and metastasis of ovarian cancer through detection of related markers. MicroRNAs (miRs) are a class of single-stranded RNAs recently found to be involved in tumor development and metastasis as well as other physiological processes and play a significant role in the diagnosis of tumor development, recurrence and metastasis due to carcinogenic or anti-carcinogenic properties.^4^ As an acknowledged cancer-promoting gene, miR-21 is highly expressed in liver and lung cancer tissues.^5^ but rarely studied in patients with ovarian cancer. Carbohydrate antigen 125 (CA125) derived from coelomic epithelium is a member of the mucin family of glycoproteins.

In recent years, CA125 has been widely used as a marker of gynecologic cancer especially useful for active surveillance because it has a high expression level in advanced and recurrent ovarian cancer.^6^ Human epididymal secretory protein 4 (HE4) is strongly associated with tumor invasion, migration and recurrence. As a new tumor marker, it is highly expressed in most ovarian cancer tissues.^7^ In this study, serum miR-21, HE4, and CA125 expression in ovarian cancer patients were measured to analyze the value of simultaneous detection of miR-21, HE4, and CA125 in predicting postoperative recurrence and metastasis of ovarian cancer.

## METHODS

A total of 169 patients who were diagnosed with ovarian conditions in Luanzhou Hospital of Traditional Chinese Medicine between January 2016 and March 2019 were enrolled in this study and assigned to a benign lesion (BL) group and an ovarian cancer (OC) group according to pathological findings. The BL group had 96 patients at the age of 28 to 62 years old, and the mean age was (51.4 ±9.5) years; the OC group consisted of 73 patients aged between 26 and 61, and the mean age was (50.1 ±8.9) years. During the same period, 120 healthy women who visited the hospital for a physical examination were recruited to serve as the control group. These healthy participants were 26 to 60 years old, and the mean age was (50.6 ±9.3) years. There was no significant age difference between the patients and the healthy women (*p*> 0.05).

### Ethical Approval:

The study was approved by the Institutional Ethics Committee of Luanzhou Hospital of Traditional Chinese Medicine on May 23, 2019(No.:2019015), and written informed consent was obtained from all participants.

### Inclusion Criteria:

- The diagnostic criteria for ovarian cancer were established by reference to the histological classification and diagnostic criteria set up by International Society of Gynecological Pathologists;^8^
- The patient had no history of ovarian cancer;
- Complete clinical information was available;
- The patient underwent both imaging tests and routine surgical treatment (laparoscopic ovarian tumorectomy).

### Exclusion Criteria:

- Immune disorders;
- Serious liver or kidney dysfunction;
- History of major cardiac surgery;
- Recently on radiotherapy or chemotherapy to treat cancer.

### This study mainly used the following reagents and instruments:

RNA extraction kit (Invitrogen, US), PrimeScript™ RT Reagent Kit with gDNA Eraser (TAKARA, Japan), miScript SYBR® Green qPCR Kit (QIAGEN, Germany), synthetic primers (Sangon Biotech, China), real-time fluorescence quantitative PCR (RT-fqPCR) system and automatic biochemical analyzer (Bio-Rad, US), HE4 ELISA Kit (Invitrogen, US), microplate reader (Bio-Rad, US), and electrochemiluminescence immunoassay analyzer (ECLIA) (Roche, Switzerland).

Pretreatment of blood samples**:** Fasting peripheral blood (3-5 mL) was drawn from each subject and centrifuged at 3000×g, 4°C for 10 min after standing at room temperature for 20 min to harvest and place serum in a centrifuge tube and refrigerated at -80°C for RNA extraction and biomarker detection.

The RNA extraction kit was used for total RNA extraction from serum according to the instructions for use, and cDNAs were obtained through reverse transcription with the miScript SYBR® Green qPCR Kit. RT-fqPCR was performed to amplify miR-21. The 20 μL qRT-PCR mixture consisted of: 10 µL of miScript SYBR® Green Mix, 2 µL of cDNAs (50 ng/µL), 1 µL of forward and reverse primers (10 µM) each, and 6.0 µL of ddH2O. Reaction conditions were: 95°C, 90 s; 95°C, 30 s; 62°C, 30 s; 72°C, 15 s; 40 cycles. Primer sequences of miR-21 and the internal control are as shown in Table-I. Quantitative analysis of serum miR-21 expression was undertaken with the 2 −∆∆CT method.

**Table-I:** Primer sequences for qRT-PCR.

miRNA	F primer 5'-3'	R primer 5'-3'
miR-21	ACGTTGTGTAGCTTATCAGACTC	AATGGTTGTCTCCACATCTC
U6	GCTTCGGCAGCACATATACTAAAAT	CGCTTCACAAATTTGCGTGTCAT

The serum level of HE4 was determined via ELISA as instructed by the ELISA kit. The serum level of CA125 was determined using ECLIA.All patients with ovarian cancer received follow-up for a year till March 2020 and underwent pathological and imaging tests to screen for recurrent or metastatic ovarian cancer. With a poor prognosis being defined as postoperative recurrence, appearance of new lesions or metastasis of lesions, the patients were assigned to the GP group and PP group according to the follow-up results.

### Statistical Analysis:

The software SPSS 25.0 was used for statistical analysis. Measurement data were expressed by “mean ± standard deviation (x̄±s)”, and intergroup comparisons were examined by the t-test (between groups) or ANOVA (≥3 groups); further, intergroup differences of statistical significance were verified by the SNK-Q test. Enumeration data were represented by n. The Pearson correlation analysis was conducted to study the correlations of serum miR-21, HE4, and CA125 expression levels with clinical characteristics of ovarian cancer patients. The ROC curve analysis was performed to investigate the diagnostic value of serum miR-21, HE4, and CA125 expression in predicting poor prognosis in ovarian cancer patients. P< 0.05 indicated a difference of statistical significance.

## RESULTS

Compared with the control group, the BL and OC groups experienced a marked increase in serum miR-21, HE4, and CA125 expression (*p*< 0.05, respectively), with the serum levels of miR-21, HE4, and CA125 in the OC group significantly higher than in the BL group (*p*< 0.05, respectively).In the ovarian cancer patients, serum miR-21, HE4, and CA125 expression levels were independent of pathological patterns (*p*> 0.05) and associated with the clinical staging, degree of transformation and lymphatic metastasis of ovarian cancer (all *p*< 0.05). Particularly, the serum levels of miR-21, HE4, and CA125 were substantially higher in patients with stage III-IV ovarian cancer showing a low degree of differentiation and lymphatic metastasis.Compared with the preoperative levels, serum miR-21, HE4, and CA125 expression in patients with ovarian cancer were significantly reduced after laparoscopic ovarian tumorectomy (*p*< 0.05, respectively).

**Fig.1:**
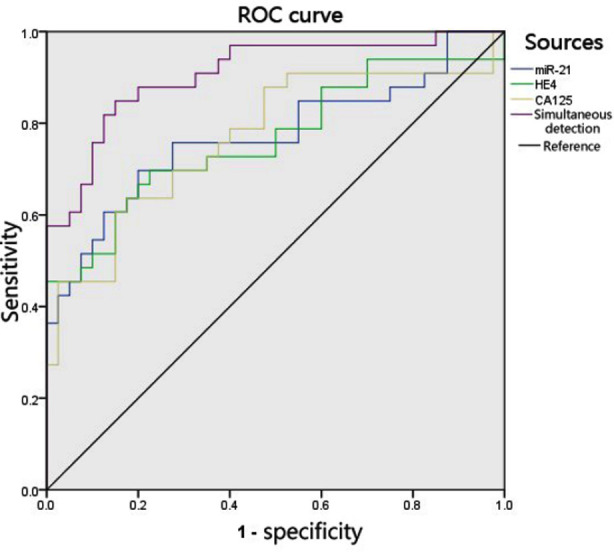
Diagnostic value of serum miR-21, HE4, and CA125 expression in predicting poor prognosis in ovarian cancer patients.

Compared with the GP group, the PP group experienced a significant increase in serum miR-21, HE4, and CA125 expression (*p*< 0.05, respectively). In terms of the three markers predictive of poor prognosis in ovarian cancer, the ROC curve analysis suggested that serum miR-21 expression had an area under the curve (AUC) of 0.769 (95% CI: 0.655-0.883), with a sensitivity of 69.7%, a specificity of 80.0% and a cutoff value of 1.536; serum HE4 expression yielded an AUC of 0.770 (95% CI: 0.656-0.895), with a sensitivity of 69.6%, a specificity of 77.5% and a cutoff value of 157.004 pmol/L; as to serum CA125 expression, the AUC was 0.764 (95% CI: 0.649-0.880), the sensitivity and specificity were 63.6% and 82.5%, and the cutoff value was 175.243 kU/L; simultaneous detection of these markers demonstrated improved diagnostic value for ovarian cancer patients at risk of poor prognosis (*p*< 0.05). Table-VI and Fig.1.

**Table-II:** Comparison of serum miR-21, HE4, and CA125 expression levels among different groups.

Group	Patients (n)	miR-21/U6	HE4 (pmol/L)	CA125 (kU/L)
Control	120	1.06±0.21	16.31±3.26	12.69±2.53
BL	96	1.85±0.37	58.66±11.73	30.16±6.03
OC	73	2.65±0.53	239.06±47.81	240.37±48.07
F-value		74.351	188.984	225.607
p-value		0.000	0.000	0.000

**Table-III:** Correlations between serum miR-21, HE4, and CA125 expression levels and clinical characteristics of ovarian cancer patients.

Group	Patients (n)	miR-21			HE4 (pmol/L)			CA125 (kU/L)		

			Χ^2^	P		Χ^2^	P		Χ^2^	P
Age			
≤50	39	2.63±0.52	0.406	0.686	238.97±47.79	0.017	0.987	239.88±47.97	0.093	0.926
>50	34	2.68±0.53			239.16±47.83			240.93±48.18		
*Clinical staging*										
I-II	16	2.45±0.29	2.058	0.043	206.07±41.21	3.111	0.003	208.66±41.73	2.975	0.004
III-IV	57	2.71±0.48			248.32±49.66			249.27±49.85		
*Pathological patterns*										
Endometrioid	11	2.64±0.40	0.018	0.982	238.81±42.22	0.001	0.999	238.89±47.07	0.017	0.983
Mucinous	12	2.63±0.50			238.60±42.34			238.86±47.71		
Serous	50	2.66±0.57			239.22±47.47			241.10±48.02		
*Degree of Differentiation*										
Moderate to high	40	2.45±0.49	3.547	0.001	230.08±37.09	2.154	0.035	232.851±32.16	2.074	0.042
Low	33	2.89±0.57			249.95±41.67			249.49±36.36		
*Lymphatic metastasis*										
No	50	2.52±0.50	3.169	0.002	233.09±32.88	2.158	0.034	234.36±35.09	2.059	0.043
Yes	23	2.94±0.58			252.06±38.82			253.42±40.20		

**Table-IV:** Comparison of serum miR-21, HE4, and CA125 expression levels in ovarian cancer patients before and after surgery.

Group	Patients (n)	miR-21/U6	HE4 (pmol/L)	CA125 (kU/L)
Preoperative	73	2.65±0.53	239.06±47.81	240.37±48.07
Postoperative	73	1.53±0.30	105.68±21.13	78.74±15.74
*t*-value		15.713	21.802	27.302
*p*-value		0.000	0.000	0.000

**Table-V:** Comparison of postoperative serum miR-21, HE4, and CA125 expression levels between GP group and PP group.

Group	Patients (n)	miR-21	HE4 (pmol/L)	CA125 (kU/L)
GP	40	1.28±0.52	118.21±39.64	126.91±45.38
PP	33	2.71±0.84	242.08±90.41	245.33±110.06
t-value		8.903	7.811	6.203
p-value		0.000	0.000	0.000

**Table-VI:** Diagnostic value of serum miR-21, HE4, and CA125 expression in predicting poor prognosis in ovarian cancer patients.

Group	AUC	Cutoff Value	Sensitivity (%)	Specificity (%)	95% CI
miR-21	0.769	1.536	69.7	80.0	0.655-0.883
HE4	0.770	157.004 pmol/L	69.6	77.5	0.656-0.895
CA125	0.764	175.243 kU/L	63.6	82.5	0.649-0.880
Simultaneous detection	0.908	-	84.8	85.0	0.838-0.977
Z1, P	2.633, 0.008				
Z2, P	2.614, 0.009				
Z3, P	2.109, 0.034				

## DISCUSSION

Ovarian cancer is a gynecologic malignancy associated with a relatively high mortality rate. Recurrence of ovarian cancer refers to the situation where ovarian cancer returns 6 months after medication is discontinued following cytoreductive surgery and chemotherapy. Recurrent ovarian cancer is associated with hereditary factors and the choice of treatment and can metastasize to the liver or the lungs, which imposes a serious threat to patient’s health.^9^,^10^ Therefore, enhanced postoperative surveillance plays a critical part in ovarian cancer treatment to timely discover recurrent signs and symptoms. By regulating the translation and expression of related genes, miRs serve as key players in numerous physiological and pathological activities, as well as the development and progression of ovarian cancer, liver cancer and many other neoplastic disorders.^11^ A high expression level of miR-21 is recorded in different types of cancer. Wang et al.^12^ showed that patients with ovarian cancer exhibited a relatively high level of serum miR-21 expression, which was associated with the clinical staging of ovarian cancer, with advanced ovarian cancer having a serum miR-21 expression level higher than early ovarian cancer. Li et al.^13^ discovered that serum miR-21 expression was higher with malignant ovarian tumors as compared with benign ones, which revealed the carcinogenic effects of miR-21 and demonstrated its diagnostic value for studies on the incidence and prognosis of ovarian cancer. Having a complex structure and a high molecular weight, CA125 exists in numerous human tissues and is highly expressed in patients with ovarian cancer. HE4 is a N-glycosylated secretory protein of small molecular weight, which is secreted by ovarian cancer cells into blood circulation, especially during the early stage.^14^ CA125 and HE4 are two tumor markers extensively used in gynecology research. Serum HE4 and CA125 are shown to have relatively high expression levels in most patients with advanced ovarian cancer, which can be reduced effectively in those who respond well to surgical treatment; however, the reduced serum HE4 and CA125 expression can be reversed if the surgical procedure fails or ovarian cancer relapses. Detection of CA125 alone is likely to yield false-positive results and has low sensitivity and specificity for ovarian cancer. Therefore, clinical diagnosis of primary or recurrent ovarian cancer usually depends on the determination of serum HE4 and CA125.^15^ Yang et al.^16^ reported satisfactory results using serum HE4 and CA125 simultaneously for the detection of early ovarian cancer. However, as the combined use of miR-21, HE4, and CA125 is rarely discussed in existing literature on postoperative recurrence of ovarian cancer, this study focused on the value of serum miR-21, HE4, and CA125 in the surveillance for postoperative recurrence and metastasis of ovarian cancer.

The results revealed that serum miR-21, HE4, and CA125 expression levels were significantly increased in the BL and OC groups when compared with the control group, and the serum levels of miR-21, HE4, and CA125 in the OC group were significantly higher than in the BL group, suggesting a strong association of aberrant miR-21, HE4, and CA125 expression with ovarian cancer attributed to the potential carcinogenic properties of these tumor markers. In ovarian cancer patients, serum miR-21, HE4, and CA125 expression levels were associated with the clinical staging, degree of transformation and lymphatic metastasis of ovarian cancer, which further indicated that miR-21, HE4, and CA125 might produce carcinogenic effects by promoting the proliferation, transformation and metastasis of cancer cells. Compared with the preoperative levels, serum miR-21, HE4, and CA125 expression were significantly reduced after the ovarian cancer patients received surgical treatment. Compared with the GP group, the PP group exhibited a significant increase in serum miR-21, HE4, and CA125 expression. This demonstrated that carcinogenic factors in ovarian cancer patients could be dramatically reduced by effective surgical treatment; besides, increased serum miR-21, HE4, and CA125 predisposed patients to recurrent or metastatic ovarian cancer. Therefore, these tumor markers were thought to have diagnostic value in predicting postoperative recurrence and metastasis of ovarian cancer. Results of the ROC curve analysis revealed that simultaneous detection of miR-21, HE4, and CA125 had a comparatively high sensitivity and specificity to diagnose poor prognosis of ovarian cancer. To be specific, ovarian cancer patients with serum miR-21 >1.536, serum HE4 >157.004 pmol/L and serum CA125 >175.243 kU/L were at higher risk for poor prognosis, under which circumstance early intervention should be made to prevent recurrence and metastasis of ovarian cancer.

### Limitations of the study:

The number of subjects included in this study is limited, so the conclusions drawn may not be very convincing. In addition, we only analyzed the cases included in our hospital, which may not be representative enough. Further studies are needed because the mechanisms responsible for the aberrant expression of these tumor markers are unclear.

## CONCLUSION

Sserum miR-21, HE4, and CA125 expression levels are strongly associated with postoperative recurrence and metastasis of ovarian cancer, and the simultaneous detection of these tumor markers shows diagnostic value in predicting poor postoperative prognosis in ovarian cancer patients.

### Authors’ Contributions:

**ZG &** **SH:** designed this study and prepared this manuscript, and are responsible and accountable for the accuracy or integrity of the work.

**CZ & HZ:** Collected and analyzed clinical data.

**JX & XS:** Significantly revised this manuscript.
